# Large-Eddy Simulation on the Aerodynamic and Thermal Characteristics in a Micropipe of the Hypersonic Engine Precooler

**DOI:** 10.3390/mi13040637

**Published:** 2022-04-17

**Authors:** Junqiang Zhang, Zhengping Zou, Yifan Wang

**Affiliations:** 1 School of Energy and Power Engineering, Beihang University, Beijing 100191, China; zhangjq6775@163.com (J.Z.); wangyifan04@buaa.edu.cn (Y.W.); 2 National Key Laboratory of Science and Technology on Aero-Engine and Aero-Thermodynamics, Beihang University, Beijing 100191, China; 3 Research Institute of Aero-Engine, Beihang University, Beijing 102206, China

**Keywords:** micropipe, supercritical methane, turbulent heat transfer, large-eddy simulation

## Abstract

The precooling air-breathing technique has become a study focus in the aerospace field. Research on the internal flow and heat-transfer mechanism of the precooler is important for design and optimization. A large-eddy simulation was used to study the aerodynamic and thermal characteristics in a micropipe of the hypersonic engine precooler with supercritical methane as coolant and fuel. Under the effect of buoyancy, the high-temperature and low-density fluid near the wall in the circumferential direction gradually accumulate to the top wall. The accumulation of low-density fluid enhances the thermal acceleration effect at the top wall, which intensifies the local turbulent relaminarization and forms an M-shaped velocity distribution, resulting in the weakening of the heat transfer. On the other hand, the high-density fluid gathers to the bottom wall under the influence of gravity, the local thermal acceleration effect is weakened, and the flow heat transfer is enhanced. The influence of the relationship between the turbulent burst and the turbulent heat transfer under the effect of buoyancy is analyzed. It is found that the low-speed ejection events and high-speed sweep events are strengthened at the bottom wall, especially the low-speed ejection. However, the occurrence of these events at the top wall is restrained to a certain extent.

## 1. Introduction

**Figure 1:**
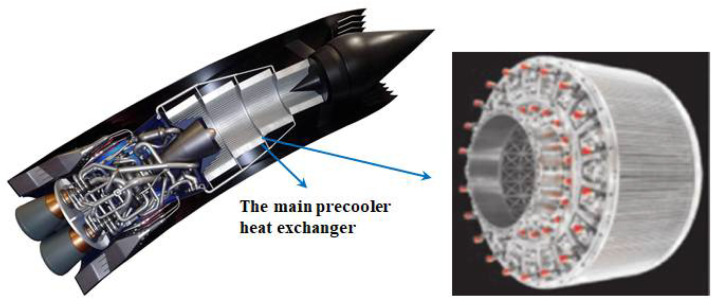
Schematic diagram of the precooled air-breathing engine [5].

During the exploration of hypersonic power, the development route of the combined-cycle power system is highly feasible, which is one of the most prospective propulsion systems in the future [1]. The high temperature of inflow air caused by hypersonic flight is a fundamental reason to limit the development of hypersonic power [2]. With the flight Mach number increasing, the stagnation temperature of air flow raises rapidly. High inlet temperature results in excess compression work and overhigh temperature at the compressor outlet, seriously limiting engine performance [3]. The hypersonic precooled engine is a potential propulsion system for hypersonic flight and space access, which can efficiently operate over a wide range of Mach numbers [4]. The research of precooled air-breathing engines with a precooler as the core component has become one of the main directions of hypersonic power exploration. By installing a precooling heat exchanger or jet precooling device at the engine inlet, as shown in Figure 1 [5], the thrust-to-weight ratio of the engine can be improved significantly, as well as the overall performance of the engine [6]. Therefore, the precooling air-breathing technique has become a study focus in the aerospace field.

Micro-and miniscale flow passages for the coolant in air precoolers have been con-centrated on thanks to their compact configuration and high heat-removal potential. However, even though microchannel heat transfer has been utilized in some vehicles (e.g., Skylon spaceplane and its SABRE), the drastically increasing heat-transfer requirements of the aerospace vehicles are still calling for extensive investigations on the heat-transfer mechanisms [7,8]. When using the hydrocarbon fuel as coolant, the flight Mach number of an existing turbojet could be expanded to 3.0–4.0 by the precooler, instead of further researching high-speed turbojets. For example, a Mach 5.0 precooled-tbcc prototype was well-tested by Hermeus Corporation, and the precooling technology ensures that a conventional turbojet successfully works at a flight condition greater than Mach 3.2. This advantage further intensifies the demand for precooler studies, especially with hydrocarbon fuel coolant [9]. Using supercritical methane as the heat-exchange medium of the precooler has many advantages. Compared with hydrogen, methane has no “hydrogen embrittlement” problem, higher operational safety, larger bulk density, and smaller loading space; compared with aviation kerosene, liquid methane as a low-temperature coolant has higher latent heat and higher calorific value. It is one of the ideal coolants for precooled expansion-cycle engines [10]. Under supercritical pressure, liquid methane enters the cooling system at a subcritical temperature. When the temperature rises across the pseudocritical point T_pc_, it becomes a supercritical fluid. Small changes in temperature and pressure will cause drastic changes in thermophysical properties, which makes the flow heat-transfer process very complicated. The generation mechanism of different heat-transfer modes has not reached a unified conclusion. Therefore, there is still a lack of in-depth understanding of supercritical methane flow and heat transfer in cooling channels, especially research on the internal flow and heat-transfer mechanism of the heat exchanger that combines the supercritical fluid and the microscale. It is of great significance to the design and optimization of the precooler.

In recent years, many scholars have carried out extensive experiments on the flow and heat-transfer characteristics of supercritical hydrocarbon fuels, including aviation kerosene, n-decane, etc. [11,12,13], but there are still few experimental studies on supercritical methane. On the other hand, with the recent advances in computing power and computational methods, numerical simulation is a powerful tool that can be used to better understand the experimental results by providing detailed information of the flow heat-transfer characteristics and to supplement data under boundary conditions that cannot be easily achieved in experiments. Pizzarelli et al. [14] showed that methane heat-transfer deterioration can occur in the regenerative cooling channels of future liquid-oxygen/liquid-methane rocket engines with chamber pressures higher than about 50 bar. The S-A turbulence model was used to analyze the heat-transfer data of supercritical methane in a heated tube for different levels of pressure, temperature, and mass flux. A Nusselt number correlation that can describe the convective heat-transfer characteristics of supercritical methane flow is proposed. The behavior of pseudocritical temperature and density versus pressure were studied and new relations were proposed by Shokri et al. [15]. The accuracy of different Nusselt relations for estimating the heat-transfer coefficient of methane at supercritical pressures was evaluated. Further, the current Nusselt relations were developed, and improved correlations were proposed for methane at the supercritical pressure inside a rectangular channel. The transient response behavior of low-temperature methane flow and heat-transfer characteristics under supercritical pressure were numerically studied by Ruan et al. [16]. The results showed that the increase in fluid temperature during heat transfer under supercritical pressure leads to a significant decrease in fluid density and intense thermal expansion of the fluid, which leads to flow oscillations. Li Hui et al. [17] systematically studied the effects of different boundary conditions on the heat-transfer characteristics of supercritical methane in microscale channels, including heat flux, mass flow rate, and system pressure. Huang et al. [18] investigated the convective heat transfer of cryogenic-propellant methane in horizontal corrugated tubes at supercritical pressures by using a standard turbulence model. The effects of several key influential parameters on both heat-transfer enhancement and pressure drop were investigated, including the pitch-to-height ratio, wall thermal conductivity, wall heat flux, inlet pressure, and Reynolds number. The performance evaluation criteria were adopted to evaluate the thermal performance influenced by these parameters. Ricci et al. [19] investigated the flow and heat-transfer characteristics of supercritical methane in cooling channels through experiments and numerical simulations. It was shown that numerical simulations can allow a detailed description of important phenomena such as thermal stratification and thermal deterioration occurring inside the channel when methane flows in transcritical conditions.

At present, many research achievements have been made on supercritical methane in the cooling channel, but the numerical calculation is mainly carried out by Reynolds-Averaged Navier-Stokes (RANS). Although the fluids under supercritical pressure remain a single phase, their transport properties near the quasi-critical temperature change drastically, which exceeds some assumptions in the derivation of the RANS model, and affects the calculation accuracy to a certain extent [20]. The RANS method can be used to analyze the flow and heat-transfer characteristics of supercritical fluid for microscale tubes in terms of time-average statistics. However, for the mechanism study, considering the flow structure changes caused by strong temperature gradient and the coupling mechanism between flow and heat transfer, a more accurate numerical simulation method is needed. In recent years, with the rapid improvement of computer capabilities, it has become possible to apply direct numerical simulation (DNS) and large-eddy simulation (LES) techniques to study the fluid dynamics and heat transfer of supercritical fluids [20,21,22,23,24,25]. Because the DNS calculation is very time-consuming, LES has been used in the numerical study of heat transfer of various supercritical fluids, showing good accuracy [21,23,24].

However, as far as the author knows, there are few reports on large-eddy simulation studies of supercritical methane flow and heat-transfer characteristics in cooling channels. In this paper, the flow and heat-transfer characteristics of methane in a horizontal micro-pipe under supercritical pressure are studied by large-eddy simulation, with emphasis on the flow and heat-transfer characteristics under the influence of buoyancy and flow acceleration. The purpose is to reveal the change in flow structure caused by a strong temperature gradient and its coupling mechanism with heat transfer.

## 2. Mathematical Model and Validation

### 2.1. Physical Model

**Figure 2:**
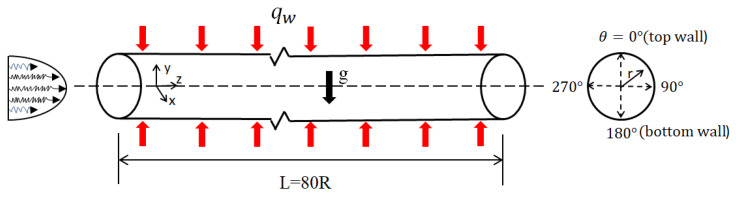
Physical model of the micropipe.

**Figure 3:**
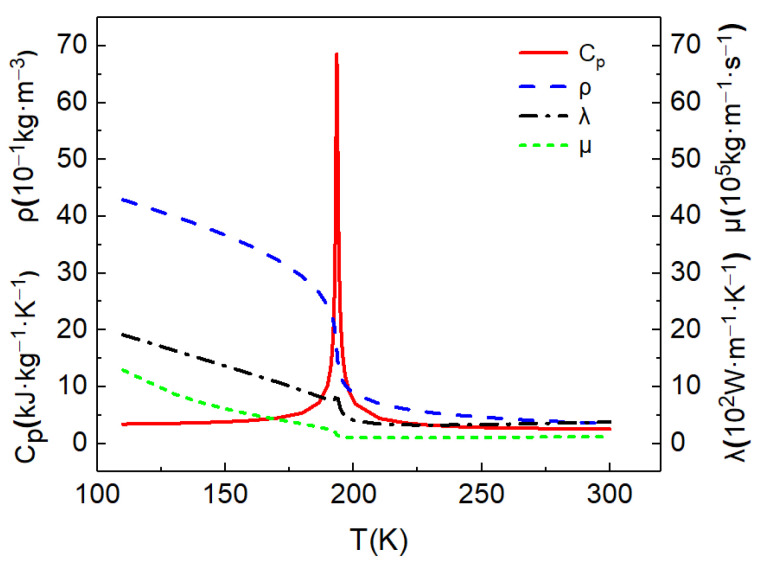
Calculated thermophysical properties of methane (5 MPa).

The computational mode was a horizontal micropipe with 80R in length (L) and 0.4 mm radius (R), as shown in Figure 2. Cryogenic liquid methane was injected into the tube from the left side at supercritical pressures. The inlet fluid temperature was T_0_ = 190 K, and the pressure was P_0_ = 5 MPa. The pipe was heated uniformly in the circumferential direction, and constant wall heat flux $q_{w}=125\;\mathrm{kW}/m^{2}$ was applied. Buoyancy-affected heat transfer at supercritical pressures is an important phenomenon. Therefore, two cases are investigated in this paper, as shown in Table 1, including mixed convection with buoyancy and forced convection without buoyancy. The physical properties of supercritical fluid change dramatically with its temperature and pressure. However, due to the small pressure drop between the inlet and outlet of the horizontal pipe, it is approximately considered that the physical properties of methane are only a function of the temperature. The specific heat capacity, density, thermal conductivity and viscosity of the low-temperature methane at 5 MPa were calculated by the REFPROP software. The physical properties were used to calculate in the FLUENT software by defining a piecewise-linear function of temperature for the properties of supercritical methane. The calculation results were fitted to the physical properties according to the linear interpolation method, and the specific results are shown in Figure 3.

### 2.2. Boundary Conditions

The junction between the fluid and the wall is set with no-slip boundary condition, and the gravity force is the negative direction of y-axis for mixed convection. The velocity-inlet type and pressure-outlet type were chosen for the inlet and outlet boundary conditions, respectively. The fully developed inlet-turbulent fluctuating-velocity field was generated by the discrete synthetic turbulence method (DSRFG).

A very important issue to ensure the obtaining of accurate LES or DNS results is to generate a random flow field as an inflow boundary condition (inflow turbulence) satisfying prescribed spatial correlations and turbulence characteristics. It is necessary to provide three-dimensional instantaneous turbulent velocity signals at the inlet of the calculation domain. This problem becomes particularly important for turbulence in spatial development, such as the flow of supercritical fluid under heating conditions. In this case, periodic boundary conditions cannot be specified, such as fully developed channel flow. Therefore, the importance of accurately simulating the inlet-turbulent flow field has been emphasized by many researchers.

Based on the synthetic random Fourier method, Smirnov et al. [26] proposed a random turbulence-generation technology, which has been widely used. Subsequently, Huang et al. [27] improved Smirnov’s method and proposed a new turbulence synthesis method named Discrete Synthetic Turbulence Method (DSRFG), which can theoretically obtain fluctuation-velocity distribution satisfying any target energy spectrum, and has been adopted by many scholars. In this paper, the DSRFG method is used to generate inlet turbulence by writing a program, and the correctness of the program is verified by calculating the working conditions in Huang’s paper. The specific formula is detailed in literature [27].

**Figure 4:**
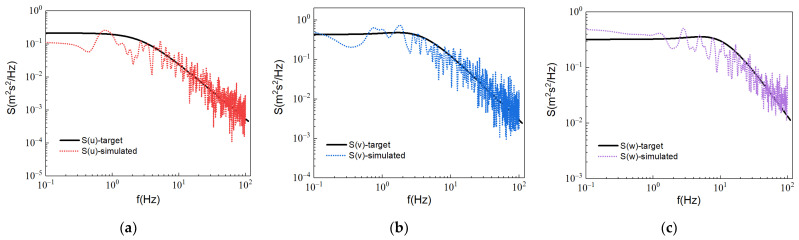
Comparison of the spectra simulated by the DSRFG with the target spectrum. (**a**) x-axis energy spectrum, (**b**) y-axis energy spectrum, (**c**) z-axis energy spectrum.

As shown in Figure 4, the calculated energy spectrum obtained by the generated fluctuation velocity is in good agreement with the target energy spectrum, indicating that the method has good processing ability for nonuniform anisotropic turbulence. The method can generate a three-dimensional transient turbulent fluctuation field satisfying the predefined energy spectrum at the inlet boundary.

### 2.3. Mathematical Model 

**Figure 5:**
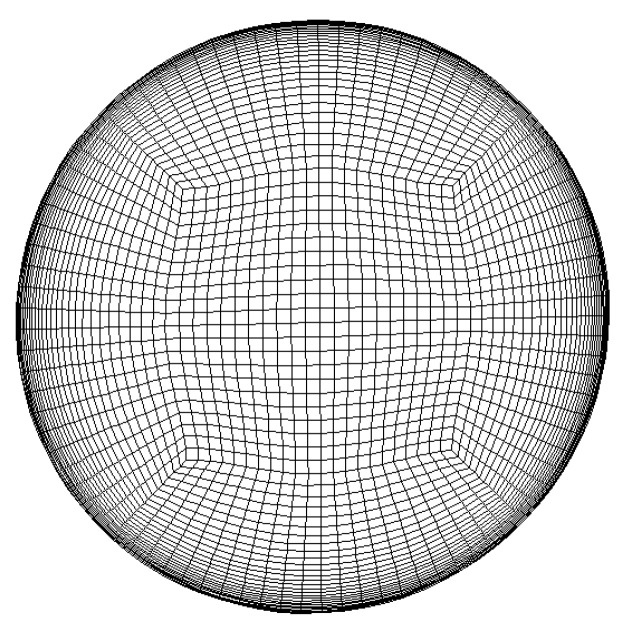
Schematic for grid.

In the current working conditions, the compressibility effect of methane under supercritical pressure is ignored and is generally regarded as incompressible flow. Under this assumption, the governing equations of mass, momentum, and energy are as follows:

(1) $$\frac{\partial \rho }{\partial t}+\frac{\partial (\rho u_{i})}{\partial x_{i}}=0$$

(2) $$\frac{\partial (\rho u_{i})}{\partial t}+\frac{\partial (\rho u_{i}u_{j})}{\partial x_{j}}=-\frac{\partial p}{\partial x_{i}}+\frac{\partial }{\partial x_{j}}[(\mu +\mu_{SGS})(\frac{\partial u_{i}}{\partial x_{j}}+\frac{\partial u_{j}}{\partial x_{i}})]+\rho g_{i}$$

(3) $$\frac{\partial (\rho h)}{\partial t}+\frac{\partial (\rho u_{j}h)}{\partial x_{j}}=\frac{\partial }{\partial x_{j}}[(\alpha +\alpha_{SGS})\frac{\partial h}{\partial x_{j}}]$$

(4) $$\mu_{SGS}=\rho \Delta^{2}\frac{{\left(S_{ij}^{d}S_{ij}^{d}\right)}^{3/2}}{{\left(\bar{S_{ij}}\bar{S_{ij}}\right)}^{5/2}+{\left(S_{ij}^{d}S_{ij}^{d}\right)}^{3/2}}$$

where ρ is the density; μ is the dynamic viscosity; h is fluid enthalpy; p is the pressure; α is the thermal diffusivity; and $u_{i}$ stands for the velocity components in x-, y-, and z-direction. In Equations (2) and (3), $\mu_{SGS}$ and $\alpha_{SGS}$ represent the SGS viscosity and SGS thermal diffusivity, respectively. The SGS viscosity is computed by using WALE model (wall-adapting local eddy viscosity) developed by Nicoud and Ducros (1999). The specific expression is as follows (see the literature for details [28]):

In this paper, the CFD software Fluent is used to study the flow and heat-transfer characteristics of methane in a horizontal micropipe under supercritical pressure, and the three-dimensional transient turbulent fluctuation velocity field is given by UDF. The velocity–pressure coupling is processed by the pressure-implicit separation operator (PISO) algorithm. The bounded second-order implicit scheme is used for temporal discretization, and the bounded central difference scheme is used for convection-term spatial discretization. In order to ensure the stability of the calculation, CFL < 1 is ensured. The time-step size used in the present study is $2\times 10^{-5}$ s. The computational grid is shown in Figure 5. In order to capture the information in the velocity-boundary layer and temperature-boundary layer, the grid height of the first layer was 0.0012 mm, and y+ < 0.25 was guaranteed. 10 layers of grid points were placed in the viscous sublayer where y+ < 5, and 21 layers in the buffer layers where y+ < 50. A mesh-independence study was conducted to identify an appropriate mesh density for the aimed calculations. In the Table 2, it can be seen that the relative error between Grid 4 and Grid 5 is 0.018%. The grid number which is set to be 3.266 million is enough to make the calculated results independent from grids.

The accuracy of the numerical method in this paper was verified by referring to the working condition of supercritical CO_2_ direct numerical simulation in the literature [29]. In this LES calculation, the energy-spectrum distribution at different radial positions (r_0_–r_3_) of the inlet cross section is directly given, as shown in Table 3, and the energy spectrum at other positions is given by interpolation, so as to achieve the three-dimensional non-uniform anisotropic-energy-spectrum distribution of the inlet. Furthermore, the fluctuating-velocity field is generated by the discrete synthetic-turbulence-method program and superimposed on the average velocity pattern obtained from DNS data.

**Figure 6:**
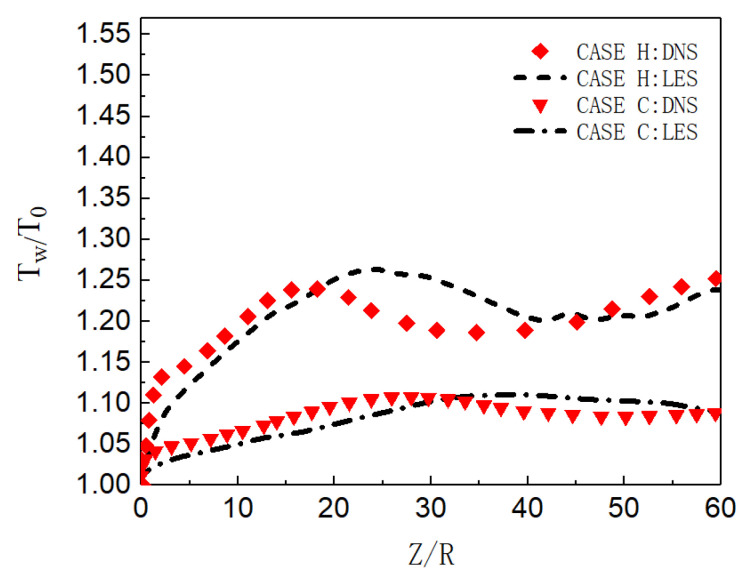
Dimensionless wall-temperature distribution.

Firstly, the time-averaged results of LES and DNS are compared, as shown in Figure 6. For both cases, the wall temperature is relatively close along the streamwise direction. Although there is a certain deviation in the distribution of wall temperature along the flow direction, it is considered that the different setting methods of inlet boundary may lead to a difference of turbulence intensity in the two flow fields, which affects the heat transfer.

**Figure 7:**
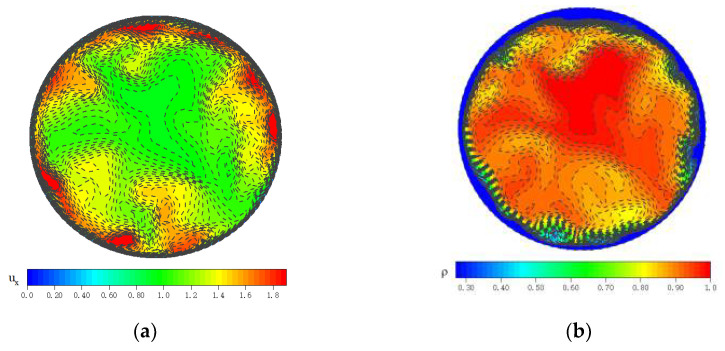
Instantaneous velocity and density distribution diagram at x = 17.5D section of case H. (**a**) velocity, (**b**) density.

Secondly, Figure 7 shows the instantaneous parameter distribution at the X = 17.5D section of case H by using LES. It can be seen that the fully developed turbulent flow field was obtained. In summary, the method of generating a fluctuating-velocity field according to the target energy spectrum at the inlet boundary and then using it for the large-eddy simulation of supercritical fluid has high calculation accuracy. It has been verified that the results are reliable for LES computations.

## 3. Results and Discussion

### 3.1. Average Statistical Characteristics

The mean quantities with Reynolds and Favre averaging are defined, where $\bar{\phi }$ is the Reynolds average of any quantity and $\tilde{\phi }=\bar{\rho \phi }/\bar{\rho }$ is the mass-weighted (Favre) average. The corresponding fluctuation components are represented by $\phi^{\prime }=\phi -\overset{-}{\phi }$ and $\phi^{\prime \prime }=\phi -\tilde{\phi }$, respectively.

**Figure 8:**
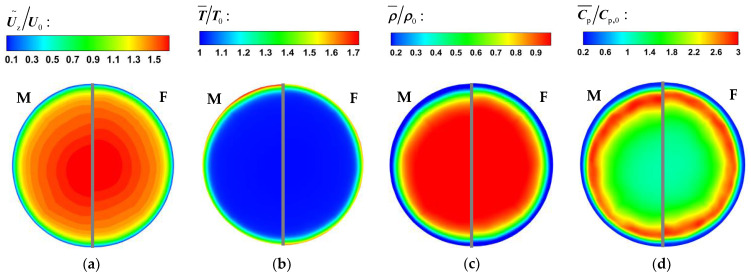
The time-averaged parameter distribution at section Z/R = 20. (**a**) velocity, (**b**) temperature, (**c**) density, (**d**) specific heat.

**Figure 9:**
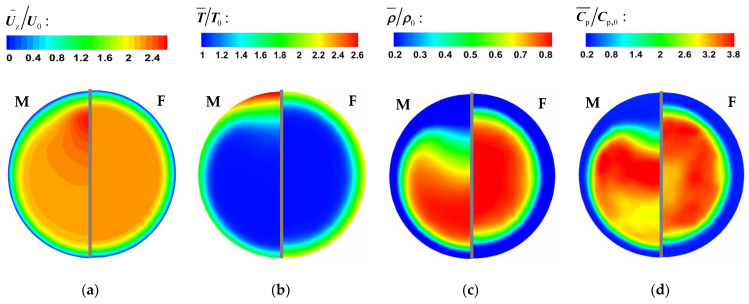
The time-averaged parameter distribution at section Z/R = 60. (**a**) velocity, (**b**) temperature, (**c**) density, (**d**) specific heat.

Figure 8 and Figure 9 show the comparison of time-averaged parameters of mixed convection (M) with buoyancy and forced convection (F) without buoyancy. The variables are dimensionless by the corresponding parameters of the inlet. It can be seen from Figure 8 and Figure 9 that the bulk time-averaged parameters of forced convection are uniformly distributed in different sections, and the wall temperature also presents a similar distribution trend along the circumferential direction. At section Z/R = 20, the distribution of time-average parameters of mixed convection is basically the same as that of forced convection. However, there is a local high-velocity region at the top wall of section Z/R = 60 in Figure 8, which is an obvious flow-acceleration phenomenon. The bulk physical properties and wall temperature are nonuniformly distributed in the circumferential direction. Compared with the bottom wall, there is an obvious high-temperature zone at the top wall, which means that the heat transfer decreases.

Supercritical fluids have a peak value of specific heat near the quasi-critical temperature, so the peak value of specific heat is often used to approximately determine the transcritical position. According to the distribution of specific heat in Figure 8d and Figure 9d, at the initial stage of flow heat transfer (Z/R = 20), a narrow high-specific-heat zone is formed in the circumferential direction near the wall. As the flow heat transfer proceeds downstream, the bulk temperature increases and the range of the high-specific-heat zone gradually expands. However, due to the significant influence of buoyancy, the high-specific-heat zone accumulates to the bottom of the circular tube, which will help to increase the local heat transport of fluid.

**Figure 10:**
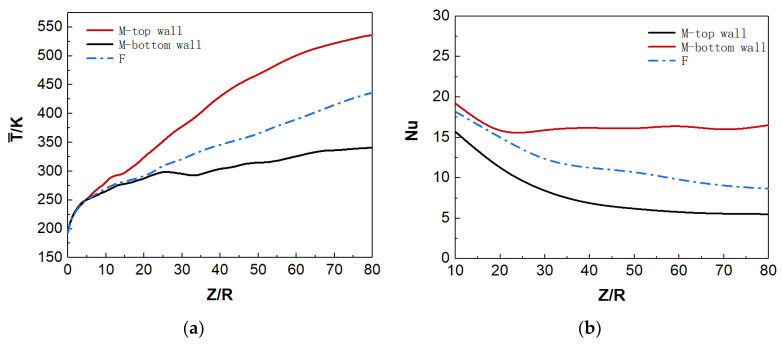
(**a**) Distribution of the wall temperature along the streamwise direction. (**b**) Distribution of the Nu along the streamwise direction.

Figure 10a shows the distribution of wall temperature and bulk temperature along the flow direction. For mixed convection, the top and bottom wall temperatures show different trends under uniform-heat flux heating. Before the position of Z/R = 10, the top and bottom wall temperatures have similar trends and values; but when Z/R > 10, the top wall temperature is significantly higher than that at the bottom, and the temperature difference gradually increases along the streamwise direction. On the other hand, according to the local Nusselt number (Nu) distribution in Figure 10b, the Nu of the top wall is small and gradually decreases along the streamwise direction, while the Nu of the bottom wall is relatively large and changes slightly along the streamwise direction. The heat-transfer deterioration occurs on the top wall to a certain extent, and the convective heat transfer is significantly weaker than that at the bottom. In addition, the wall temperature and Nu in the forced convection are between the corresponding parameters of the top wall and the bottom wall in the mixed convection, indicating that the buoyancy has a significant effect on the convection heat transfer, and the effect is different at different spatial positions.

**Figure 11:**
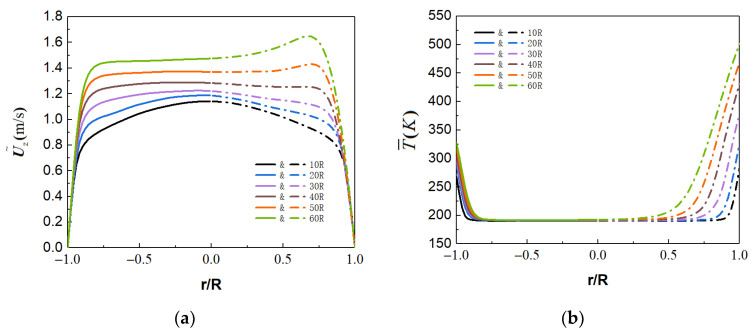

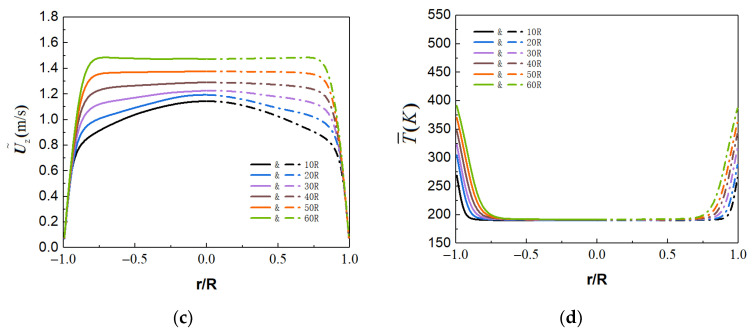
(**a**) Streamwise velocity and (**b**) fluid temperature for mixed convection. (**c**) Streamwise velocity and (**d**) fluid temperature for forced convection. Solid lines for θ=0° and dotted lines for θ=180°.

Figure 11c,d show the time-averaged velocity and bulk temperature distribution of the forced convection along the streamwise direction. These average parameters are uniformly distributed in the radial direction, which has similar distribution laws in different sections. However, there are significant differences in mixed convection. Figure 11a shows the radial distribution of streamwise velocity at different sections of mixed convection. At the initial stage of fluid heat transfer, the flow velocity is approximately symmetrical at the section of Z/R = 10, indicating that the initial change of fluid physical properties at this position has little effect on the flow. As the fluid flows downstream, the physical properties of the fluid tend to change dramatically, and the flow structure of the flow field in the microtube also changes. The flow velocity presents an asymmetric distribution, especially the M-shaped velocity distribution is formed at the top wall of sections Z/R = 50 and Z/R = 60. It makes the flow in that region significantly faster than in the bulk region. This asymmetry is more significant in the average temperature distribution, as shown in Figure 11b. Within the same section, the fluid-temperature gradient at the top (Y/R = 1) is significantly smaller than that at the bottom (Y/R = −1). The increase of temperature boundary-layer thickness leads to the decrease in heat transfer in the top region.

**Figure 12:**
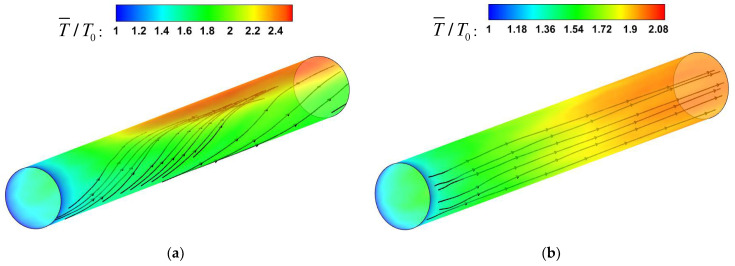
Three-dimensional streamline diagram. (**a**) Mixed convection, (**b**) forced convection.

The area where the fluid physical properties change rapidly is mainly concentrated near the wall, so it would be more intuitive to analyze the effect of buoyancy force through the three-dimensional streamlines diagram near the wall. In mixed convection, as shown in Figure 12a, the wall temperature of the micropipe is nonuniformly distributed in the circumferential direction, and there is a local high-temperature zone at the top wall. Under supercritical pressure, liquid methane enters the micropipe from the inlet in a horizontal direction. The streamlines near the wall flow horizontally near the inlet. With the increase in buoyancy, the streamlines begin to tilt upward, and the tilt angle gradually increases. The streamlines flow horizontally near the inlet. With the increase in buoyancy, the streamlines begin to incline upward, and the inclination angle gradually increases. Note that the streamlines incline upward along the curved surface, which is very close to the wall, not in the flow core area. In the whole circumferential direction, the low-density fluid near the wall continues to gather at the top. On the other hand, the low-temperature and high-density fluid accumulates at the bottom wall under the influence of gravity. The accumulation of fluids of different densities will have an effect on the local flow acceleration.

**Figure 13:**
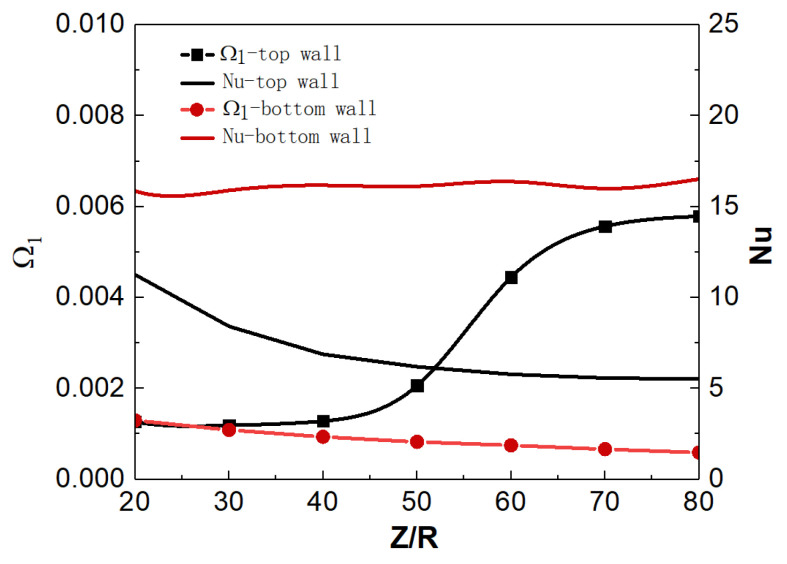
Relationship between flow-acceleration parameters $\Omega_{1}$ and Nu.

The effect of flow acceleration is typically represented by parameter $\Omega_{1}$ which is defined as $\Omega_{1}=\frac{du_{b}}{dx}\frac{D}{u_{b}}=\frac{4\beta q_{w}}{\rho c_{p}u_{b}}$. HE [30] suggests that $\Omega_{1}$ is a suitable parameter for correlating the data from their convection heat transfer of supercritical fluid. The heat-transfer impairment increases with increasing $\Omega_{1}$. For the present simulations, as shown in Figure 13, the Nu decreases with the increase in $\Omega_{1}$ along the streamwise direction. The increase in $\Omega_{1}$ represents the enhancement of the flow acceleration effect. It is inferred that the accumulation of low-density fluid at the top enhances the local flow-acceleration effect, which intensifies the local turbulent laminarization, resulting in the weakening of the heat transfer of the top wall. This also explains the reason for the M-type velocity distribution of the fluid at the top wall in Figure 11a. At the bottom wall, Nu increases with the decrease in $\Omega_{1}$ along the streamwise, but the increase is limited. Under the influence of gravity, the low-temperature and high-density fluid gathers at the bottom wall, and the local flow-acceleration effect is weakened. This process will be beneficial to the enhancement of the convection heat-transfer performance. However, as shown in Figure 12b, the streamlines near the wall are approximately horizontally distributed in forced convection, and the wall-temperature distribution is relatively uniform in the circumferential direction. The effect of buoyancy on the flow structure will be analyzed in the following sections.

### 3.2. Turbulence Characteristics

**Figure 14:**
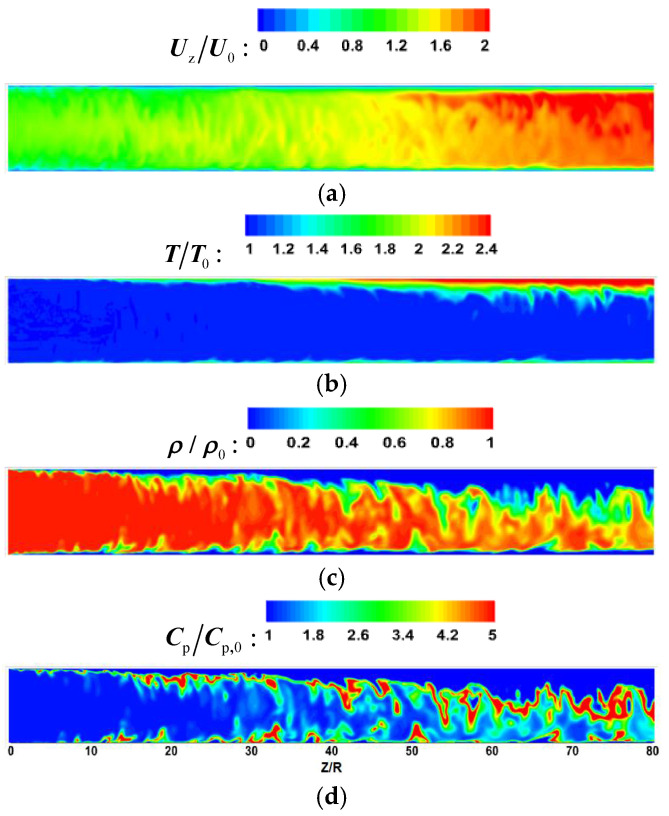
The instantaneous parameter distribution at section x = 0 for mixed convection. (**a**) velocity, (**b**) temperature, (**c**) density, (**d**) specific heat capacity.

**Figure 15:**
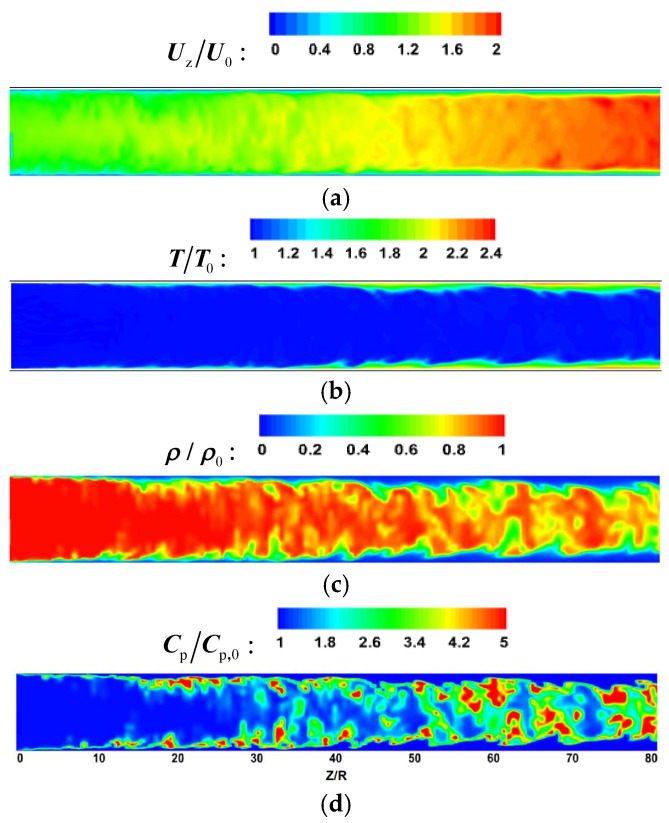
The instantaneous parameter distribution at section x = 0 for forced convection. (**a**) velocity, (**b**) temperature, (**c**) density, (**d**) specific heat capacity.

Figure 14 and Figure 15 show the dimensionless transient flow characteristics in the micropipe, and the convective heat transfer is in a state of strong turbulence. In the forced convection of Figure 15, although the flow parameters and physical parameters of the streamwise direction change drastically at different spatial positions, they all show an approximately uniform distribution at different axial positions. For the mixed convection, according to the instantaneous velocity distribution of Figure 14a, the fluid-acceleration effect at the top wall is stronger than that at the bottom, especially at the middle and rear of the pipe. In Figure 14b, there is a significant difference in the turbulent heat transfer between the top wall and bottom wall. In the middle and rear of the micropipe, more high-temperature fluids gather near the top under the action of buoyancy. As shown in Figure 14c,d, the fluid with high density and high specific heat gradually gathers towards the bottom wall during the flow process, which increases the instantaneous heat transfer of the bottom wall.

With the progress of heating, the fluid temperature increases continuously along the flow direction. The fluid-temperature-rise rate near the wall is high, so the fluid in this area first reaches the supercritical temperature T_pc_. The drastic change in fluid physical properties leads to the change of flow-field flow structure, which affects the heat-exchange process.

**Figure 16:**
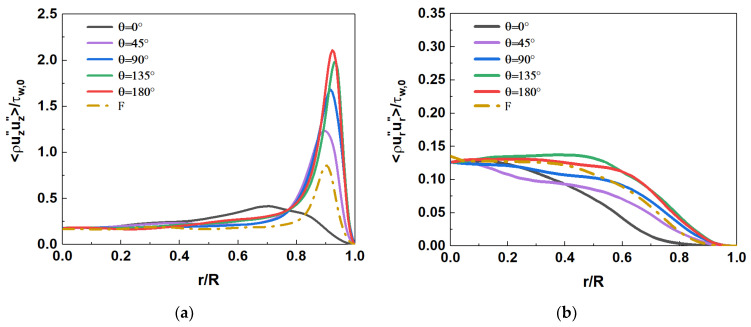
Reynolds stress distribution at the section of Z/R = 50. (**a**) axial Reynolds stress $\bar{\rho U_{z}^{\prime \prime }U_{z}^{\prime \prime }}/\tau_{w,0}$, (**b**) radial Reynolds stress $\bar{\rho U_{r}^{\prime \prime }U_{r}^{\prime \prime }}/\tau_{w,0}$.

Figure 16 shows the comparison of axial and radial Reynolds stress distribution at section Z/R = 50. In mixed convection, the distribution of axial Reynolds stress $\bar{\rho U_{z}^{\prime \prime }U_{z}^{\prime \prime }}/\tau_{w,0}$ is quite different in the circumferential direction. Near the top of the wall (θ=0°), the axial Reynolds stress decreases sharply, so that the single peak almost disappears. The axial Reynolds stress increases gradually with the downward direction along the circumference. A local peak appears near the wall, and the position of the peak gradually approaches the wall. The axial Reynolds stress reaches its maximum when it reaches the bottom wall (θ=180°). The radial Reynolds stress $\bar{\rho U_{r}^{\prime \prime }U_{r}^{\prime \prime }}/\tau_{w,0}$ shows a similar variation pattern, but there is no local peak near the wall. Compared to forced convection (F), the axial Reynolds stress $\bar{\rho U_{z}^{\prime \prime }U_{z}^{\prime \prime }}/\tau_{w,0}$ is enhanced in the range of circumferential angle θ≈45°–180°. The radial Reynolds stress $\bar{\rho U_{r}^{\prime \prime }U_{r}^{\prime \prime }}/\tau_{w,0}$ decreases in the range of θ≈0°–90°, while it increases significantly in the range of θ≈90°–180°. It shows that the influence of buoyancy on turbulent motions at different spatial positions is different. It will be further explained in combination with the distribution of turbulent kinetic energy in the following sections.

**Figure 17:**
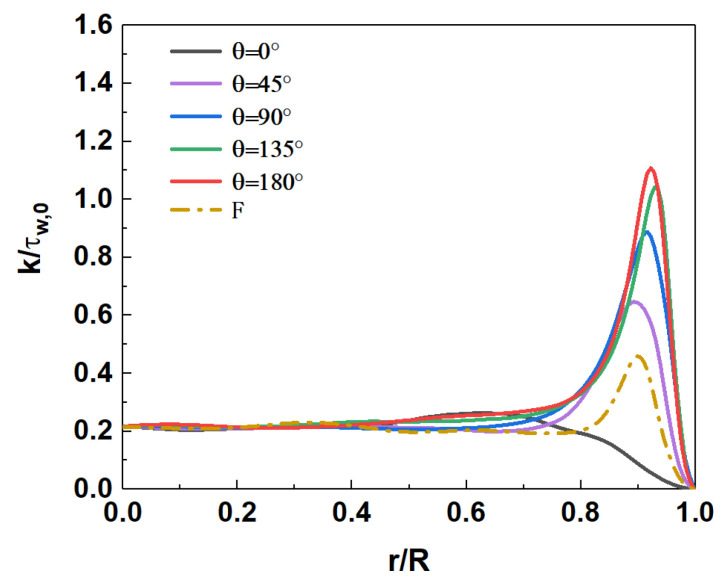
Distribution of turbulent kinetic energy $k/\tau_{w,0}$ at the section of Z/R = 50.

Figure 17 shows the distribution of dimensionless turbulent kinetic energy $k/\tau_{w,0}$ at section of Z/R = 50. For the mixed convection, the turbulent kinetic energy gradually increases from the top wall downward along the circumference, and reaches the maximum at the bottom wall. Compared with the forced convection, the turbulent kinetic energy at the bottom wall of the mixed convection increases to about 2.2 times, while the turbulent kinetic energy at the top wall decreases to 0.5 times.

**Figure 18:**
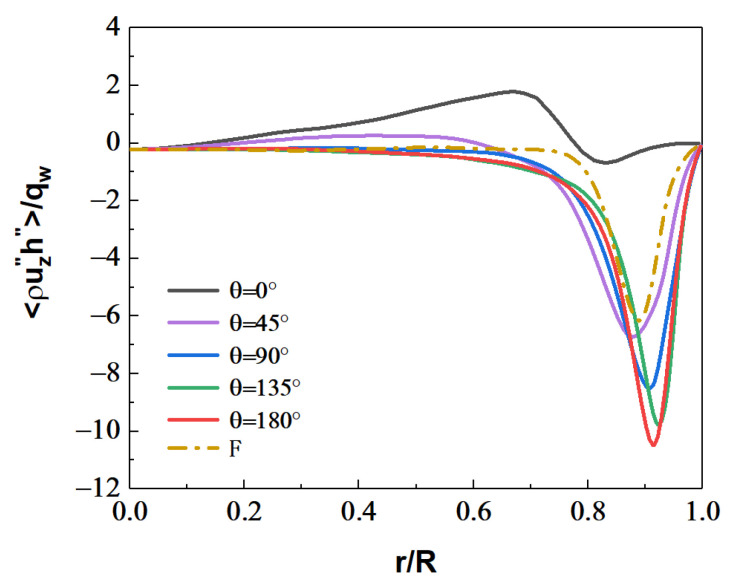
Distribution of turbulent heat fluxes $\bar{\rho U_{z}^{''}h^{\prime \prime }}/q_{w}$ at the section of Z/R = 50.

Figure 18 shows the distribution of turbulent heat fluxes $\bar{\rho U_{z}^{\prime \prime }h^{\prime \prime }}/q_{w}$ along the streamwise direction. The turbulent heat fluxes near the wall have a similar trend to the turbulent kinetic energy near the wall along the circumferential direction. Near the top wall, the turbulent kinetic energy is reduced, and the corresponding turbulent heat-transfer performance is weakened. However, the turbulent kinetic energy reaches a maximum near the bottom wall, and the corresponding turbulent heat fluxes are also the maximum.

**Figure 19:**
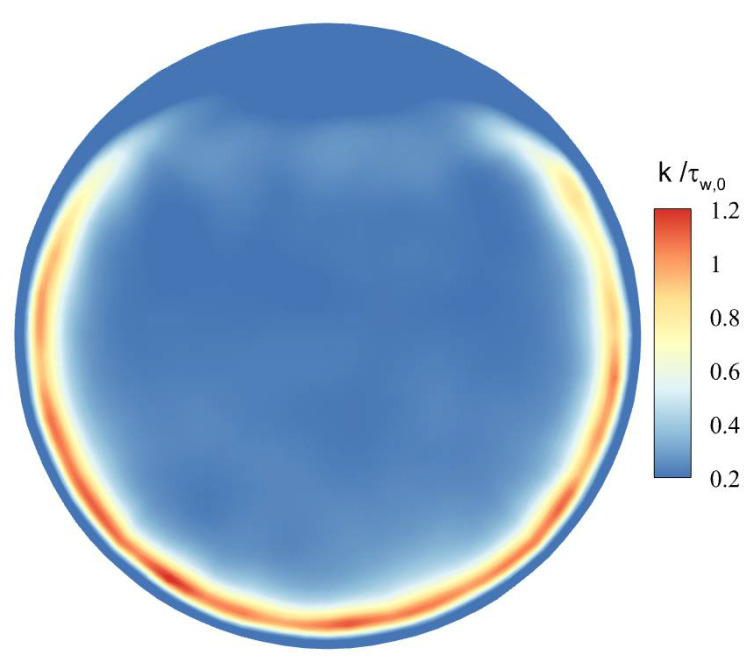
The contour of turbulent kinetic energy $k/\tau_{w,0}$ at the section of Z/R = 50.

**Figure 20:**
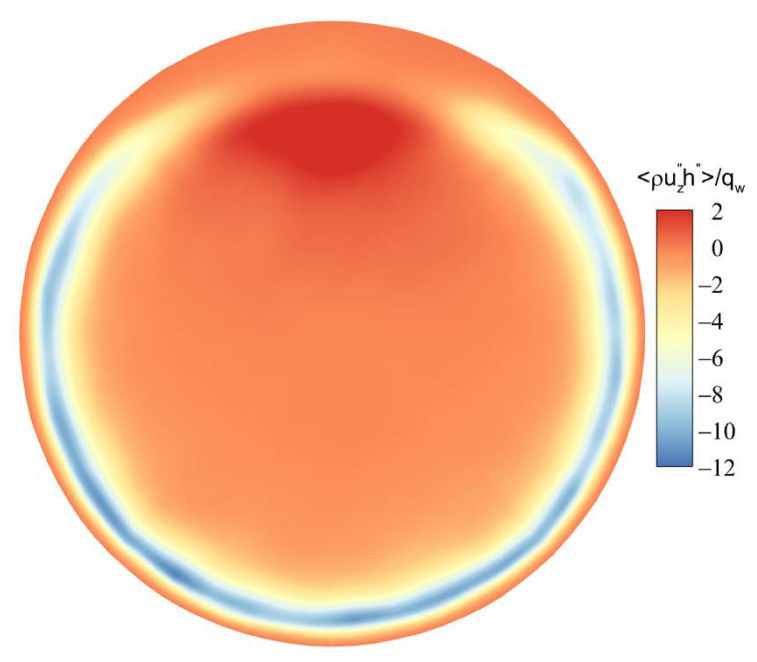
The contour of turbulent heat fluxes $\bar{\rho U_{z}^{\prime \prime }h^{\prime \prime }}/q_{w}$ at the section of Z/R = 50.

Figure 19 and Figure 20 shows the distribution of turbulent kinetic energy and turbulent heat fluxes at the section of Z/R = 50. The turbulent kinetic energy and turbulent heat transfer are enhanced in the circumferential-angle range of θ≈45°–315°, indicating that the turbulence suppression is mainly concentrated in a certain angle range at the top, rather than intuitively believing that the turbulence of the upper semicircular tube is suppressed and the lower semicircular tube is strengthened. On the other hand, it can be found that there is an obvious positive correlation between turbulent kinetic energy and turbulent heat fluxes. In order to deeply study the interaction mechanism between the two, the relationship between the turbulent burst behavior near the wall and the turbulent heat transfer under the action of buoyancy is analyzed.

**Figure 21:**
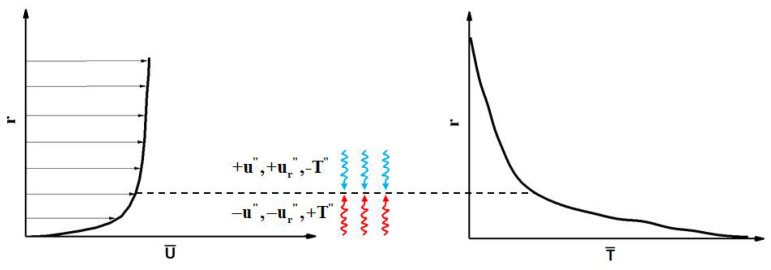
Particle motions in the wall normal direction.

Figure 21 shows the variation diagram of fluctuation velocity and fluctuation temperature corresponding to the average velocity and average temperature distribution near the wall boundary layer, so as to explain the physical process corresponding to the sign of fluctuation velocity and fluctuation temperature. As shown in Table 4, the ${\langle u^{\prime \prime }T^{\prime \prime }\rangle }_{i}$ and ${\langle u_{r}{}^{\prime \prime }T^{\prime \prime }\rangle }_{i}$ heat fluxes are classified into octants according to the signs of the fluctuations of the velocity components and temperature about their respective mean values [31]. It should be noted that the positive direction of $u_{r}{}^{\prime \prime }$ is from the center of the pipe to the wall, and the negative direction is from the wall to the center. The turbulent burst behavior near the wall is quantitatively analyzed.

Obviously, as shown in Figure 21, for a horizontal micropipe, the turbulence behavior corresponding to the sign of the fluctuation component is the same for the top wall (θ=0°) and the bottom wall (θ=180°). The main turbulent behaviors of the pipe wall are represented by octants 4 and octants 6, where octants 4 represents the sweep of high-speed and low-temperature fluid and octants 6 represents the ejection of low-speed and high-temperature fluid. The flow behaviors represented by other serial numbers in the octants are weak. In wall turbulent heat transfer, the ejection behavior represents the heat transfer from the near-wall fluid to the mainstream, and the sweep behavior represents the heat transfer from the mainstream to the near wall. The changes of these behaviors have a very important impact on the turbulent heat transfer near the wall. For mixed convection and forced convection, the turbulent heat fluxes are calculated into octants. By comparing their relative sizes, the differences of turbulent behavior with or without buoyancy are quantitatively analyzed, which affect the heat-transfer process.

**Figure A1:**
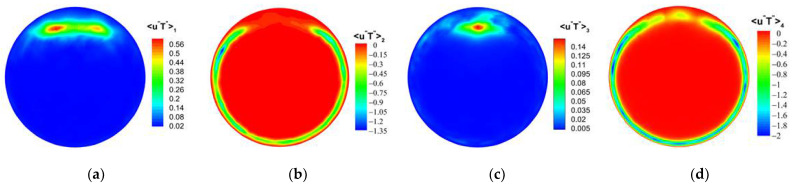

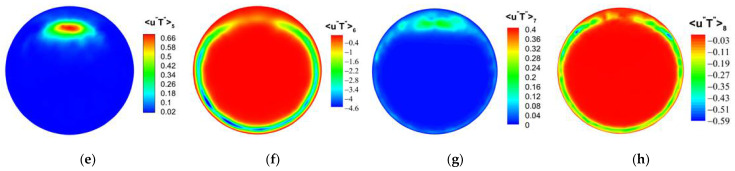
Streamwise heat fluxes ${\langle u^{\prime \prime }T^{\prime \prime }\rangle }_{i}$ from the octants for mixed convection at the section of Z/R = 50. (**a**) ${\langle u^{\prime \prime }T^{\prime \prime }\rangle }_{1}$, (**b**) ${\langle u^{\prime \prime }T^{\prime \prime }\rangle }_{2}$, (**c**) ${\langle u^{\prime \prime }T^{\prime \prime }\rangle }_{3}$, (**d**) ${\langle u^{\prime \prime }T^{\prime \prime }\rangle }_{4}$, (**e**) ${\langle u^{\prime \prime }T^{\prime \prime }\rangle }_{5}$, (**f**) ${\langle u^{\prime \prime }T^{\prime \prime }\rangle }_{6}$, (**g**) ${\langle u^{\prime \prime }T^{\prime \prime }\rangle }_{7}$, (**h**) ${\langle u^{\prime \prime }T^{\prime \prime }\rangle }_{8}$.

**Figure A2:**
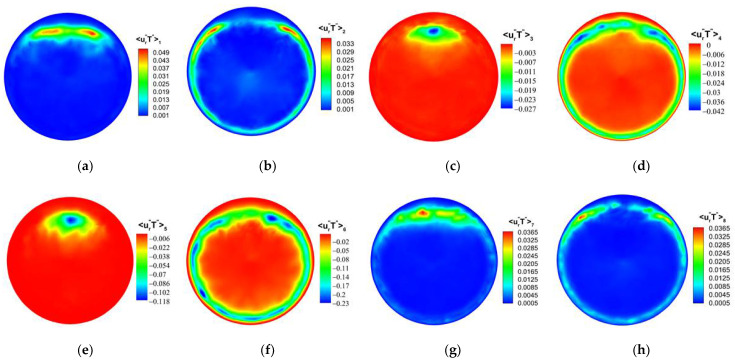
Normal heat fluxes ${\langle u_{r}{}^{\prime \prime }T^{\prime \prime }\rangle }_{i}$ from the octants for mixed convection at the section of Z/R = 50. (**a**) ${\langle u_{r}{}^{\prime \prime }T^{\prime \prime }\rangle }_{1}$, (**b**) ${\langle u_{r}{}^{\prime \prime }T^{\prime \prime }\rangle }_{2}$, (**c**) ${\langle u_{r}{}^{\prime \prime }T^{\prime \prime }\rangle }_{3}$, (**d**) ${\langle u_{r}{}^{\prime \prime }T^{\prime \prime }\rangle }_{4}$, (**e**) ${\langle u_{r}{}^{\prime \prime }T^{\prime \prime }\rangle }_{5}$, (**f**) ${\langle u_{r}{}^{\prime \prime }T^{\prime \prime }\rangle }_{6}$, (**g**) ${\langle u_{r}{}^{\prime \prime }T^{\prime \prime }\rangle }_{7}$, (**h**) ${\langle u_{r}{}^{\prime \prime }T^{\prime \prime }\rangle }_{8}$.

**Figure A3:**
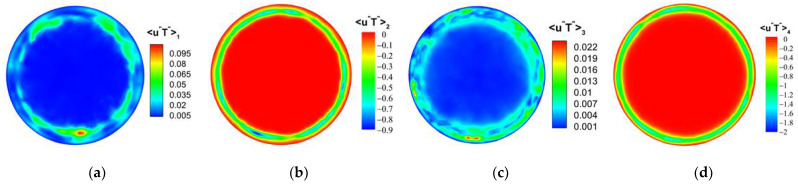

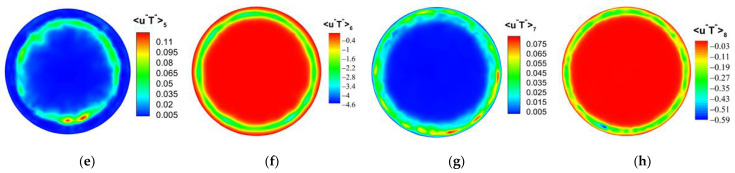
Turbulent heat fluxes ${\langle u^{\prime \prime }T^{\prime \prime }\rangle }_{i}$ from the octants for forced convection at the section of Z/R = 50. (**a**) ${\langle u^{\prime \prime }T^{\prime \prime }\rangle }_{1}$, (**b**) ${\langle u^{\prime \prime }T^{\prime \prime }\rangle }_{2}$, (**c**) ${\langle u^{\prime \prime }T^{\prime \prime }\rangle }_{3}$, (**d**) ${\langle u^{\prime \prime }T^{\prime \prime }\rangle }_{4}$, (**e**) ${\langle u^{\prime \prime }T^{\prime \prime }\rangle }_{5}$, (**f**) ${\langle u^{\prime \prime }T^{\prime \prime }\rangle }_{6}$, (**g**) ${\langle u^{\prime \prime }T^{\prime \prime }\rangle }_{7}$, (**h**) ${\langle u^{\prime \prime }T^{\prime \prime }\rangle }_{8}$.

**Figure A4:**
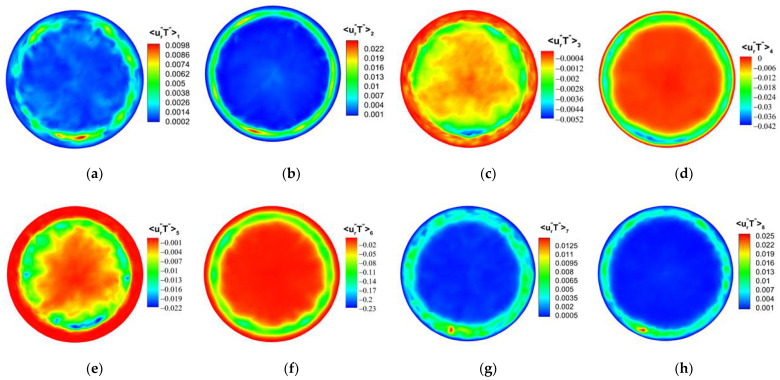
Normal heat fluxes ${\langle u_{r}{}^{\prime \prime }T^{\prime \prime }\rangle }_{i}$ from the octants for forced convection at the section of Z/R = 50. (**a**) ${\langle u_{r}{}^{\prime \prime }T^{\prime \prime }\rangle }_{1}$, (**b**) ${\langle u_{r}{}^{\prime \prime }T^{\prime \prime }\rangle }_{2}$, (**c**) ${\langle u_{r}{}^{\prime \prime }T^{\prime \prime }\rangle }_{3}$, (**d**) ${\langle u_{r}{}^{\prime \prime }T^{\prime \prime }\rangle }_{4}$, (**e**) ${\langle u_{r}{}^{\prime \prime }T^{\prime \prime }\rangle }_{5}$, (**f**) ${\langle u_{r}{}^{\prime \prime }T^{\prime \prime }\rangle }_{6}$, (**g**) ${\langle u_{r}{}^{\prime \prime }T^{\prime \prime }\rangle }_{7}$, (**h**) ${\langle u_{r}{}^{\prime \prime }T^{\prime \prime }\rangle }_{8}$.

The streamwise turbulent heat fluxes ${\langle u^{\prime \prime }T^{\prime \prime }\rangle }_{i}$ and the normal turbulent heat fluxes ${\langle u_{r}{}^{\prime \prime }T^{\prime \prime }\rangle }_{i}$ at the section of Z/R = 50 are calculated, where the M-type velocity distribution appears. By comparing the values of heat fluxes ${\langle u^{\prime \prime }T^{\prime \prime }\rangle }_{i}$ and ${\langle u_{r}{}^{\prime \prime }T^{\prime \prime }\rangle }_{i}$ from the octants, as shown in Figure A1, Figure A2, Figure A3 and Figure A4, the main turbulent behaviors of the micropipe are represented by octants 4 and octants 6, and the flow behaviors represented by other serial numbers in the octants are weak. Therefore, the turbulent heat-transfer behaviors of the octants 4 and octants 6 are mainly analyzed.

**Figure 22:**
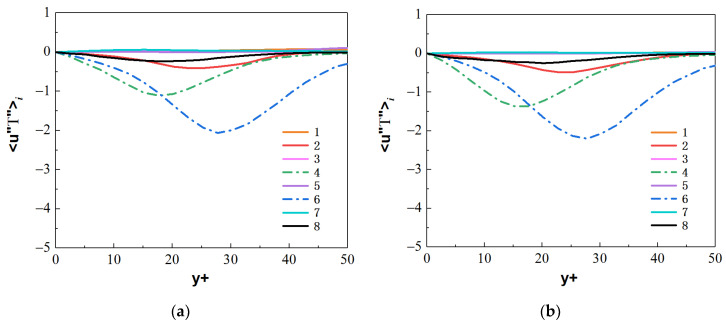
Heat fluxes ${\langle u^{\prime \prime }T^{\prime \prime }\rangle }_{i}$ from the octants for forced convection at the section of Z/R = 50. (**a**) bottom wall, (**b**) top wall.

**Figure 23:**
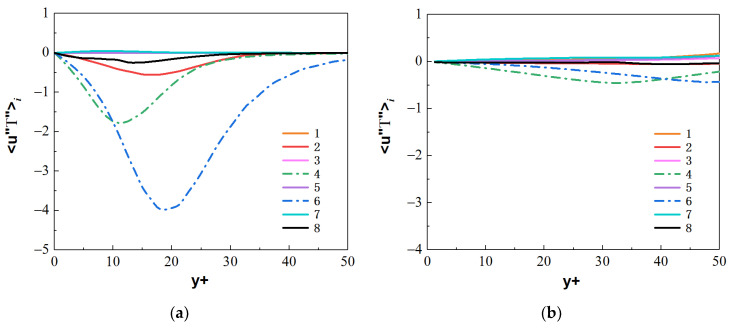
Heat fluxes ${\langle u^{\prime \prime }T^{\prime \prime }\rangle }_{i}$ from the octants for mixed convection at the section of Z/R = 50. (**a**) bottom wall, (**b**) top wall.

**Figure 24:**
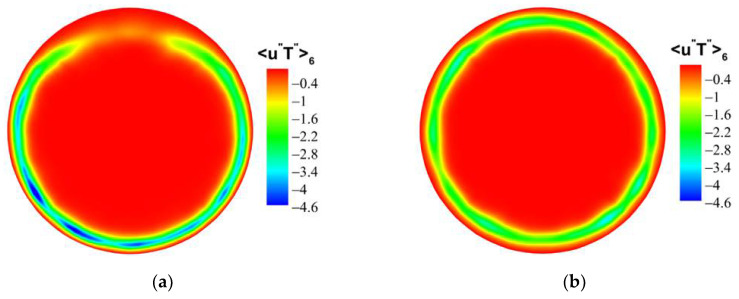
Heat flux ${\langle u^{\prime \prime }T^{\prime \prime }\rangle }_{6}$ distribution of octants 6 at the section of Z/R = 50. (**a**) mixed convection, (**b**) forced convection.

**Figure 25:**
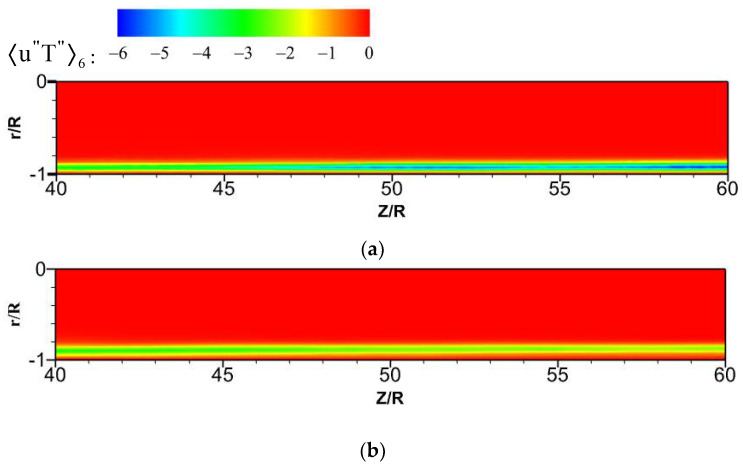
Heat flux ${\langle u^{\prime \prime }T^{\prime \prime }\rangle }_{6}$ distribution of octants 6 along the streamwise direction at the bottom wall (θ=180°). (**a**) mixed convection, (**b**) forced convection.

For the forced convection, as shown in Figure 22, the octants 4 (green dotted line) and octants 6 (blue dotted line) of the bottom/top wall correspond to strong turbulence behavior. The value of octants 6 is significantly larger than that of octants 4. It shows that the ejection motion is stronger than the sweep motion, and the intensity of the same type of motion on the top and bottom walls is almost equal. As shown in Figure 23, the turbulent motion in mixed convection appears to be very different. At the bottom wall, the turbulence behaviors corresponding to octants 4 and octants 6 have been significantly enhanced; in particular, the ejection motion corresponding to octants 6 is most obvious. However, at the top wall, the corresponding turbulent motion is very weak, indicating that the turbulent behavior is restrained. These differences are fully reflected in the comparison of mixed convection and forced convection for octants 6 at section of Z/R = 50, as shown in Figure 24. Take the heat flux ${\langle u^{\prime \prime }T^{\prime \prime }\rangle }_{6}$ distribution of octants 6 at the bottom wall (θ=180°) for the mixed convection and the forced convection as an example, which is shown in Figure 25. It can be seen that turbulence behavior corresponding to octants 6 of mixed convection has been strengthened along the streamwise direction. More low-speed and high-temperature fluid is carried from the wall to the fluid core, and the turbulent heat flux ${\langle u^{\prime \prime }T^{\prime \prime }\rangle }_{6}$ is significantly stronger than that of forced convection.

**Figure 26:**
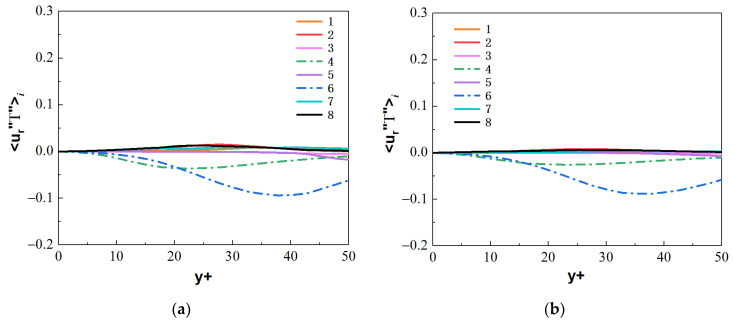
Heat fluxes ${\langle u_{r}{}^{\prime \prime }T^{\prime \prime }\rangle }_{i}$ from the octants for forced convection at the section of Z/R = 50. (**a**) bottom wall, (**b**) top wall.

**Figure 27:**
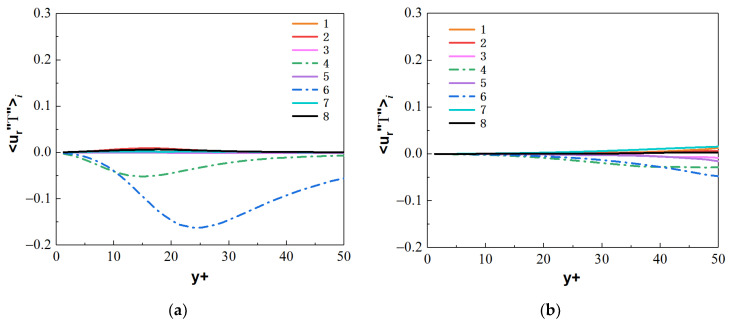
Heat fluxes ${\langle u_{r}{}^{\prime \prime }T^{\prime \prime }\rangle }_{i}$ from the octants for mixed convection at the section of Z/R = 50. (**a**) bottom wall, (**b**) top wall.

**Figure 28:**
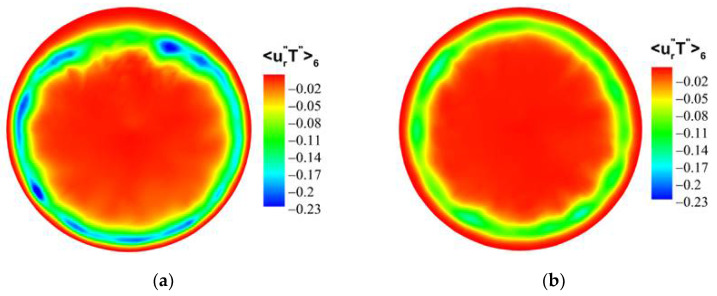
Heat-flux ${\langle u_{r}{}^{\prime \prime }T^{\prime \prime }\rangle }_{6}$ distribution of octants 6 at the section of Z/R = 50. (**a**) mixed convection, (**b**) forced convection.

**Figure 29:**
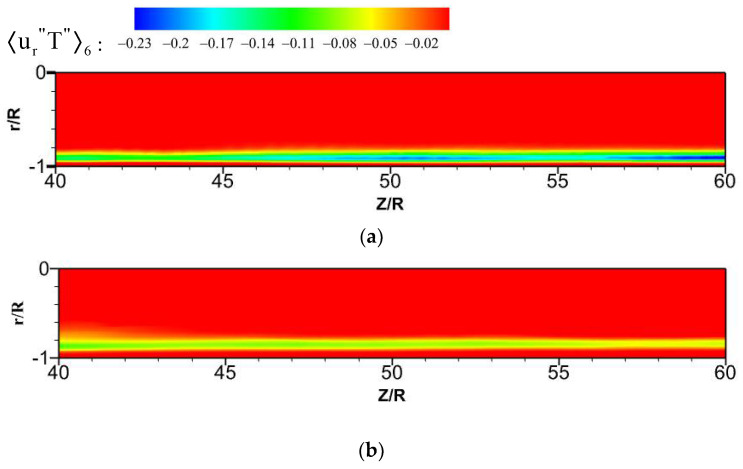
Heat-flux ${\langle u_{r}{}^{\prime \prime }T^{\prime \prime }\rangle }_{6}$ distribution of octants 6 along the streamwise direction at the bottom wall (θ=180°). (**a**) mixed convection, (**b**) forced convection.

Similarly, compare the normal turbulent heat fluxes ${\langle u_{r}{}^{\prime \prime }T^{\prime \prime }\rangle }_{i}$. As shown in Figure 26 and Figure 27, compared with the forced convection, the turbulent heat-transfer behaviors of the mixed convection corresponding to octants 4 and octants 6 at the bottom wall are strengthened, while the behaviors of octants 4 and octants 6 at the top wall are weak. Figure 28 shows the normal heat-flux ${\langle u_{r}{}^{\prime \prime }T^{\prime \prime }\rangle }_{6}$ distribution of octants 6 at the section of Z/R = 50 for the mixed convection and forced convection. In the forced convection, the normal heat flux ${\langle u_{r}{}^{\prime \prime }T^{\prime \prime }\rangle }_{6}$ is uniformly distributed along the circumferential direction. In the mixed convection, the value of the normal heat flux ${\langle u_{r}{}^{\prime \prime }T^{\prime \prime }\rangle }_{6}$ at the bottom wall is larger than that of forced convection, while the value of the normal heat flux at the top wall is less than that of forced convection at the corresponding position. Turbulent behavior is affected by buoyancy, which leads to the difference of turbulent heat transfer. Figure 29 shows the heat-flux ${\langle u_{r}{}^{\prime \prime }T^{\prime \prime }\rangle }_{6}$ distribution of octants 6 along the streamwise direction for the mixed convection and the forced convection. The turbulent heat flux ${\langle u_{r}{}^{\prime \prime }T^{\prime \prime }\rangle }_{6}$ of mixed convection is also stronger than that of forced convection along the streamwise direction.

These results show that the influence of buoyancy for convection heat transfer in the 0.8 mm diameter horizontal micropipe is still strong for high heat fluxes, when Re = 6150. Under the action of buoyancy, the flow structure varies in different spatial locations. The turbulent bursting behavior at the bottom wall is enhanced, thereby enhancing the heat transport between the fluid near the wall and the mainstream, and the heat transfer is improved. However, these behaviors are suppressed to a certain extent at the top wall, resulting in a decrease in the local convective heat transfer.

## 4. Conclusions

In this paper, the discretizing and synthesizing random-flow-generation (DSRFG) technique was used to generate three-dimensional instantaneous inflow turbulence with predefined characteristics according to the real turbulence energy spectrum. The flow and heat-transfer characteristics of methane in a horizontal micropipe under supercritical pressure are studied by large-eddy simulation, with emphasis on the flow and heat-transfer characteristics under the influence of buoyancy and flow acceleration. Under the current calculation conditions, the main conclusions obtained are as follows:

(1) At medium Reynolds number (Re = 6150) and high heat fluxes ($q_{w}=125\;\mathrm{kW}/m^{2}$), the thermophysical properties of the supercritical methane in a horizontal micropipe change rapidly, resulting in the nonuniform distribution of wall temperature along the circumferential direction. Under the effect of buoyancy, the high-temperature and low-density fluid near the wall in the circumferential direction gradually accumulate to the top wall. The accumulation of low-density fluid enhances the thermal acceleration effect of the fluid at the top wall, which intensifies the local turbulent laminarization and forms an M-shaped velocity distribution at the top wall, resulting in the weakening of the heat transfer. On the other hand, the low-temperature and high-density fluid gather to the bottom wall under the influence of gravity, the local thermal acceleration effect is weakened, and the flow heat transfer is enhanced.

(2) The change of turbulent kinetic energy near the wall at different flow sections has a significant effect on the heat-transfer process. The turbulent kinetic energy at the top wall is reduced, the corresponding turbulent heat transfer is weakened, and the heat transfer is deteriorated. The turbulent kinetic energy at the bottom wall is maintained at a high level, and the corresponding turbulent heat-transfer capacity is stronger.

(3) At medium Reynolds number and high heat fluxes, the buoyancy and thermal acceleration effect of supercritical fluid is still obvious in the flow of horizontal micropipes (D = 0.8 mm), and the turbulent burst behavior is changed, which in turn affects the local convective heat transfer. The performance is as follows: the low-speed ejection events and high-speed sweep events are strengthened at the bottom wall, especially the low-speed ejection. However, the occurrence of these events at the upper wall is restrained to a certain extent.

## Figures and Tables

**Table 1:** Operating parameters of the simulated cases.

Case	Type	g	T_0_ (K)	P_0_ (MPa)	$q_{w}\;(\mathrm{kW}/m^{2})$
1	Mixed convection	9.8	190	5.0	125
2	Forced convection	0	190	5.0	125

**Table 2:** The check of mesh independence.

No.	Cells (Million)	T_w,ave_ (K)
Grid 1	1.958	313.869
Grid 2	2.432	314.082
Grid 3	2.784	314.657
Grid 4	3.266	315.214
Grid 5	3.896	315.271

**Table 3:** The given position of the energy spectrum.

Radial Positions	Value
r_0_/D	0
r_1_/D	0.2748
r_2_/D	0.4679
r_3_/D	0.4997

**Table 4:** Classification based on the signs of fluctuation velocity and fluctuation temperature.

Octants	1	2	3	4	5	6	7	8
$u^{\prime \prime }$	+	−	−	+	+	−	−	+
$u_{r}{}^{\prime \prime }$	+	+	+	+	−	−	−	−
$T^{\prime \prime }$	+	+	−	−	+	+	−	−

## Abbreviations

**Nomenclature**	**Value**
p	pressure (MPa)
T	temperature (K)
T_w,ave_	average wall temperature(K)
h	enthalpy (J/kg)
q_w_	wall heat flux (kw/m^2^)
U	velocity (m/s)
y+	wall dimensionless distance
g	gravitational acceleration (m/s^2^)
S	energy spectrum (m^2^s^2^/Hz)
f	frequency (Hz)
Nu	Nusselt number (${\mathrm{Nu}=\mathrm{hD}/\lambda }_{b}$)
k	turbulent kinetic energy
λ	thermal conductivity (W/(m K))
μ	viscosity (kg/(m s))
ρ	density (kg/m^3^)
Cp	specific heat at constant pressure (J/(kg K))
θ	circumference angle
ϕ	physical variable
**Subscripts**	
0	inlet
w	wall
b	bulk fluid
**Symbols**	
‘¯’	Reynolds average
‘~’	mass-weighted average
‘<>’	statistical average
